# Nano-in-Micro Dual Delivery Platform for Chronic Wound Healing Applications

**DOI:** 10.3390/mi11020158

**Published:** 2020-02-01

**Authors:** Jana Zarubova, Mohammad Mahdi Hasani-Sadrabadi, Lucie Bacakova, Song Li

**Affiliations:** 1 Department of Bioengineering, University of California, Los Angeles, CA 90095, USAmmhs@ucla.edu (M.M.H.-S.); 2 Department of Biomaterials and Tissue Engineering, Institute of Physiology of the Czech Academy of Sciences, Prague 14220, Czech Republic; lucie.bacakova@fgu.cas.cz

**Keywords:** chronic wounds, angiogenesis, antibacterial properties, drug delivery, microfluidics, nanoparticles, microparticles

## Abstract

Here, we developed a combinatorial delivery platform for chronic wound healing applications. A microfluidic system was utilized to form a series of biopolymer-based microparticles with enhanced affinity to encapsulate and deliver vascular endothelial growth factor (VEGF). Presence of heparin into the structure can significantly increase the encapsulation efficiency up to 95% and lower the release rate of encapsulated VEGF. Our in vitro results demonstrated that sustained release of VEGF from microparticles can promote capillary network formation and sprouting of endothelial cells in 2D and 3D microenvironments. These engineered microparticles can also encapsulate antibiotic-loaded nanoparticles to offer a dual delivery system able to fight bacterial infection while promoting angiogenesis. We believe this highly tunable drug delivery platform can be used alone or in combination with other wound care products to improve the wound healing process and promote tissue regeneration.

## 1. Introduction

Chronic wounds are one of the common complications of ischemia, diabetes mellitus, or chronic venous insufficiency. Non-healing wounds affect about 3 to 6 million people in the United States, and result in enormous health care expenditures of more than $3 billion per year [1,2]. Normal wound healing is a dynamic process that can be divided into four major phases that include hemostasis, inflammation, proliferation, and remodeling [3]. Following vascular constriction and fibrin clot formation, the inflammatory phase begins with neutrophils and macrophages cleaning the invading microorganisms and cellular debris. Macrophages then undergo a phenotypic transition to a reparative state that promotes tissue regeneration and the transition to the proliferative phase. During this phase, fibroblasts migrate to the wound and synthesize collagen, and epithelial cells proliferate and close the wound. The formation of new blood vessels by angiogenesis plays a key role in this process by providing cells with nutrients and oxygen necessary for the developing tissue [4]. When the robust proliferation and extracellular matrix (ECM) synthesis is over, wound healing enters the final remodeling phase, in which vascular density returns to the normal level and ECM architecture approaches that of the normal tissue. 

However, in case of chronic wounds, this physiological healing process is impaired, the inflammation phase is prolonged, and the wound is susceptible to bacterial infections [5]. It is presumed that the inability of the wound to heal is caused, at least partially, by the insufficient angiogenesis [6]. For example, the levels of vascular endothelial growth factor A (VEGF-A) in wounds of diabetic mice are significantly decreased compared to normal healthy controls [7], and the diabetic mice treated with VEGF-A exhibit accelerated wound closure [8]. Although the delivery of exogenous growth factors have beneficial effects on wound healing in animal models, a majority of growth factor-based agents for wound healing have not shown the expected benefits to patients in clinical trials [6,9,10]. One of the drawbacks of these studies seems to be the lack of the system for controlled delivery and localization [10] of the growth factors to the site of injury, and the other one is a rapid degradation of growth factors in the body. For example, the biological half-life of VEGF administered intravenously was determined to be less than 30 min [11]. Thus, the development of new delivery systems that would protect growth factors from rapid degradation and that would be able to control the spatial and temporal presentation of growth factors is essential for improvement of growth factor therapeutic effects. In order to control and tune the formation of VEGF delivery carriers, we utilized microfluidic droplet generation platforms. These systems offer microscale control over the flow to manipulate high-throughput fabrication of microparticles with a wide range of physical properties [12]. This method also offers high efficiency encapsulation of active biologicals (cells and proteins) in microparticles for a variety of biomedical applications. Here, we are not only interested in developing new carriers to locally deliver angiogenetic agents, but we also consider using the same platform to deliver antibiotics. Bacterial infection is considered a major problem in chronic wounds as it can delay healing and cause patient morbidity [13]. Topical or systematic administration of antibiotics can manage the infection, but they may cause allergic reactions and/or antibiotic resistance without significant contribution to the healing process [14,15]. Utilizing sustained and localized delivery methods can improve the therapeutic outcome [16,17]. Moreover, to enhance the effectiveness of tissue repair, co-delivery of antibiotics and growth factors can be considered. Here, we developed a combinatorial microscale delivery platform able to fight bacterial infection and promote angiogenesis. This platform can be topically applied or sprayed on the wound alone or in combination with other wound care materials. Sustained release can promote the healing process while preventing bacterial infections. 

## 2. Materials and Methods

### 2.1. Chemical and Biologicals

Unless noted otherwise, all chemicals used in this study were purchased from Sigma-Aldrich, Inc. (St. Louis, MO, USA). All cell culture reagents, solutions, and dishes were obtained from Thermo Fisher Scientific (Waltham, MA, USA), except those indicated otherwise. 

### 2.2. Polymer Modification

High α-L-guluronic acid residues (G blocks) alginate (G content: 67 mol%; molecular weight: 200 kDa; FMC Biopolymer, Rockland, Maine) was oxidized (2 mol%) via controlled chemical reaction with sodium periodate, as previously reported [18]. Alginate-heparin conjugation was done according to a previously published protocol [19]. Briefly, alginate and heparin were dissolved separately in 2-(N-morpholino)ethanesulfonic acid (MES) buffer (0.05 M, pH 5.4) in a concentration of 20 mg/mL overnight at room temperature. Carboxylic acid groups of alginates (in MES: 0.05 M, pH 5.4) were activated using 1-ethyl-3-(3-dimethylaminopropyl)carbodiimide/N-hydroxysuccinimide (EDC/NHS; 0.6:1) for 15 min before addition of 31 mM ethylenediamine under vigorous stirring. Then, 4 h later heparin activated with EDC/NHS was added and reacted overnight at room temperature prior to being dialyzed (Molecular weight cut off: 8–14 kDa; Spectrum) for 3 days. The solution was then concentrated, lyophilized, and kept at −20 °C before further use.

### 2.3. Microfluidic Synthesis of Microparticles

A hydrophobic coated glass microfluidic droplet junction chip (100 µm; Dolomite Microfluidics, Charlestown, MA, USA) was utilized to make monodispersed alginate-based microparticles. An alginate-heparin solution (1.2% w/v) was used as the inner aqueous phase. Mineral oil containing 10 wt % Span 80 was used as the continuous phase to help the formation of droplets. Flow rates of 2, 3, 5, 7, and 8 μL min^−1^ for the alginate flow and 25, 15, 12, 10, 7, and 5 μL min^−1^ for oil flow were applied using two syringe pumps (Harvard Apparatus PHD 2000). Bright field images were taken at various time points using a Leica DMIL inverted microscope. The formed microparticles were collected in a 200 mM calcium ion bath and left for 20 min for ionic crosslinking prior to being extensively washed with a 10 mM NaCl solution and centrifuged (21,000 rpm for 7 min) twice before further use. 

### 2.4. Growth Factor Loading

To prepare VEGF-loaded microparticles, VEGF at different concentrations (1, 10, 50, and 100 ng/mL) was incubated with 100 µL of microparticles (1 mg/mL) in phosphate buffered saline (PBS) buffer supplemented with bovine serum albumin (BSA; 0.1%w/v) overnight at 4 °C under gentle shaking. The microparticles were then centrifuged (21,000 rpm for 7 min) and washed twice with PBS supplemented with calcium ions (10 mM CaCl_2_) to remove unabsorbed VEGF. The concentration of VEGF in the supernatant was measured using enzyme-linked immunosorbent assay (ELISA) to estimate the encapsulation efficiency (binding capacity) of alginate and alginate-heparin microparticles. Growth factor encapsulation efficiency was determined by subtracting the amount of VEGF remaining in solution for microparticle samples from growth factor solutions that lacked microparticles. Encapsulation efficiency = (total VEGF added − non-entrapped VEGF in solution) divided by the VEGF concentrations in the samples without microparticles.

### 2.5. Antibacterial Drug Loading and Nanoparticles Synthesis

Gentamicin-loaded poly(lactic-co-glycolic) acid (PLGA) nanoparticles (NPs) were prepared using a water-in-oil-in-water (W/O/W) double emulsification evaporation process, as previously reported [20,21,22]. Resomer RG 503 PLGA (50:50; molecular weight: 28 kg/mol) was used in this study. A total of 5 mg of gentamicin was dissolved in 100 µl of miliQ water, and 100 µl of 2% w/v polyvinyl alcohol (PVA) was added to this W phase. This solution was added dropwise to O phase under ultrasonication (3 min, 40%) on ice. This oil phase, which contained 100 mg of PLGA, was dissolved in 8 mL of dichloromethane. To form W/O/W, the W/O suspension was added dropwise to 25 mL of 1% w/v PVA solution and vigorously stirred overnight to allow dichloromethane evaporation. Formed NPs were centrifugated and washed before lyophilization for 2 days. Hydrodynamic diameter and surface charge of formed PLGA NPs was studied using dynamic light scattering (DLS) and zeta potential measurements (Zetasizer Nano, Malvern, UK). In order to load these NPs into alginate-based microparticles, gentamicin-loaded PLGA NPs were mixed with alginate-heparin prior to running them through microfluidic chip for droplet generation. 

### 2.6. Drug Release Study

In vitro release of gentamicin or VEGF was studied by incubating 10 × 10^6^ microparticles in 1 mL PBS (pH 7.4; supplemented with 10 mM CaCl_2_) at 37 °C. At different time intervals, 500 µL of the supernatant was separated from microparticles, centrifuged, and replaced with an equivalent volume of fresh calcium-modified PBS solution. Here, it should be noted that the presence of CaCl_2_ is essential to prevent decrosslinking of ionically gelled alginate. Although this concentration is higher than the physiological calcium concentration, it is essential for alginate gel to maintain its integrity. The concentration of released gentamicin was determined by measuring the fluorescence intensity (λ_ex_: 360 nm; λ_em_: 460 nm) of gentamicin after 2× dilution in 400 mM boric acid at pH 9.7 and derivatization with orthophthaldehyde as described previously [21,23]. The concentration of released VEGF was determined using a human VEGF ELISA kit as a function of time.

### 2.7. Antibacterial Functional Assay

*Pseudomonas aeruginosa* and *Escherichia coli* were grown in LB broth for 24 h at 37 °C. Actively growing inoculum was diluted to an optical density of 0.3 at absorption wavelength of 600 nm (A_600_) and then further diluted to 2 × 10^5^ colony-forming units (CFU) per ml. The minimum inhibitory concentration (MIC) was determined for both bacteria challenged with either free gentamicin, blank NPs, or gentamicin-loaded NPs formulations. The lowest concentration of challenge at which no observable growth was apparent after 24 h was reported as the MIC. 

### 2.8. In Vitro Angiogenesis Functional Assay

Human umbilical vein endothelial cells (HUVEC) were cultured in the Endothelial Growth Medium-2 (EGM-2, Lonza), and cells in the passage 2–5 were used in this study. Some of the experiments were performed with HUVECs stably transduced with LifeAct-Green Fluorescent Protein (GFP). Spheroids (500 cells per spheroid) were formed with the use of an AggreWell 400 device (STEMCELL Technologies, Vancouver, Canada) according to the manufacturer’s instructions. After a 24 h incubation, the spheroids were harvested and embedded in the growth factor-reduced Matrigel (Corning, New York, NY, USA) within a µ-Slide Angiogenesis chamber (Ibidi, Gräfelfing, Germany). EGM-2 medium containing microparticles was added to the samples after Matrigel gelation. Endothelial cell sprouting was monitored for up to 3 days. Alternatively, single cell HUVECs were seeded on top of the Matrigel and the effect of microparticles on capillary network formation in 2D was evaluated after 24 h. Image analysis was performed using ImageJ and the Sprout Morphology plugin [24]. We estimated the sprouting features including sprout number, length, and density of endothelial cells grown in a bead sprouting assay. Formation of cellular capillary-like networks was evaluated with the Angiogenesis Analyzer ImageJ plugin [25]. 

## 3. Results and Discussion

**Figure 1:**
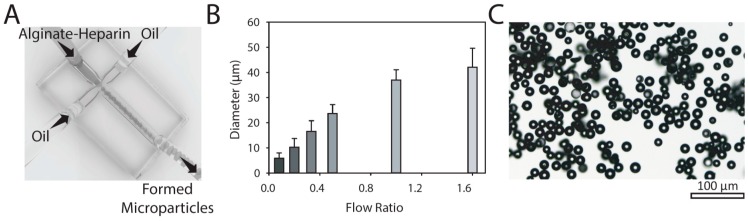
Formation of alginate-based microparticles. (**A**) Schematic of microfluidics platform used to control formation of microparticles. Hydrophobic coated glass microfluidic droplet junction chip with 100 µm diameter used in this study. (**B**) Controlling flow rates was used to tune the size of alginate-based microparticles. The presented data are expressed as average ± SD (number of independent experiments; *n* = 3; for each independent experiment more than 40 microparticles were measured). (**C**) Bright-field image of calcium crosslinked alginate-heparin microparticles at water phase/oil phase flow ratio of 0.33 (oil flow rate: 12 µL/min; water flow rate: 4 µL/min).

Here, we utilized microfluidic technology to control the formation of polysaccharide microparticles. Microfluidic platforms have recently attracted great attention due to their possibility of better controlling physical properties of formed particles including their size, shape, and dispersity [26,27,28,29,30,31]. In the current study, a microfluidic droplet generator was used (Figure 1A). Alginate biopolymers were used due to their biocompatibility and ease-of-use properties [32,33]. Engineering of microparticles was possible by changing the polymer type, concentration, flow characteristics, and crosslinking conditions. On the basis of previously published reports, we chose a concentration of 1.2% wt/v alginate and 200 mM CaCl_2_ as optimal crosslinking concentrations. Flow rates of 2–8 and 5–25 μL min^−1^ were used as alginate and oil flow rates, respectively. Figure 1B shows the effect of flow conditions on the diameter of formed particles. A microscope image of crosslinked microparticles at flow ratio of 0.33 is shown in Figure 1C. This image shows monodisperse hydrogel particles using the droplet generation technique. In this research, 16 μm particles formed at a flow ratio of 0.33 were selected for further investigation, as they were in the same size range as the cells which prevented their update. On the basis of our previous report, we also found that these particles can penetrate and distribute into porous microenvironments, which will be limited for the larger particles (>20 μm) [28]. Moreover, particles at these sizes cannot penetrate through endothelial junctions and leak into the blood vessels, which guarantee their local effect.

**Figure 2:**
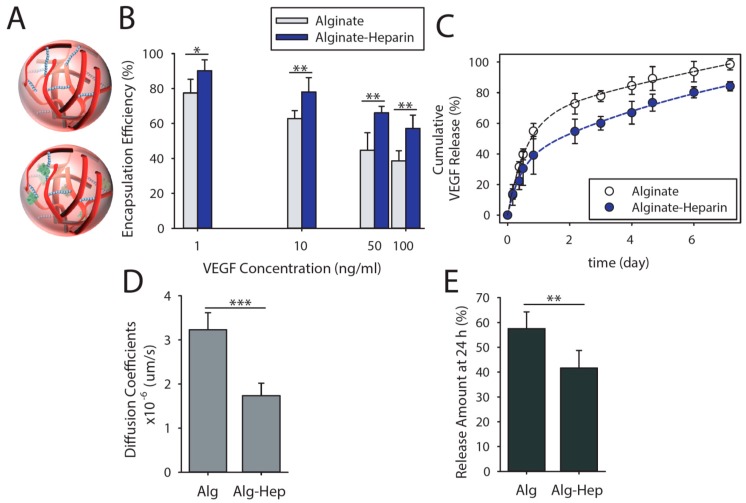
The presence of heparin increased vascular endothelial growth factor (VEGF) binding and prolongs its release. (**A**) Schematic of microparticles (16 µm in diameter) were used to encapsulate therapeutic proteins (here VEGF). (**B**) VEGF binding efficiency of alginate and alginate-heparin microparticles at various initial concentrations of VEGF after 16 h incubation at 4 °C. (**C**) VEGF release kinetic of microfluidic-synthesized microparticles prepared from alginate and alginate-heparin in phosphate buffered saline (PBS) at 37 °C. (**D**) Calculated diffusion coefficients of VEGF from microparticles and (**E**) the cumulative amount of released VEGF after 24 h. The presented data are expressed as an average ± SD; number of independent experiments, *n* = 3; number of samples per experiment, s = 5). The lines in the graphs are guide for the eye. *, **, and *** indicate significant difference of *p* < 0.05, *p* < 0.01, and *p* < 0.001, respectively, as evaluated by one-way ANOVA.

Recently, we reported the chemical modification of alginate with heparin, which increased the binding affinity of a wide variety of cytokines and growth factors [19]. The amount of conjugated heparin was determined using a toluidine blue assay, as reported previously [34]. Here, we used the alginate-heparin conjugate with 0.5 nmol of heparin per milligram of alginate, which optimally encapsulated a series of cationic proteins including interlukin-2 (IL-2), transforming growth factor β (TGF-β), interferon-gamma (IFN-g), and stromal cell-derived factor-1 alpha (SDF-1a), with isoelectric points of 7.7, 8.9, 9.5, and 9.9, respectively. The intrinsic affinity of VEGF to heparin has been reported previously [35]. Cationic features of VEGF increased the affinity of this growth factor to the alginate matrix. VEGF can be encapsulated inside alginate-heparin microparticles during the particle formation process, which can provide very high encapsulation efficiency (95% at 10 ng/mL of initial content). However, due to the loss of particles during the washing steps, we decided to use post-formation loading to reduce the cost of resulted VEGF-loaded microparticles. Particles were incubated with VEGF overnight at 4 °C at different initial concentrations, and the plotted encapsulation efficiency is shown in Figure 2B. Presence of heparin not only increased the VEGF uptake but also prolonged its release, as can be seen in Figure 2C. We also calculated the diffusion coefficient of VEGF from alginate and alginate-heparin microparticles Here, we assumed the release was solely diffusion-based (no degradation of gels during the release period) and the release kinetic was Fickian. Thus, the release data were fitted on *M_t_/M_∞_* = *kt^n^*, where *M_t_/M_∞_* represents the fraction of released VEGF at time *t*, *k* is the release constant, and *n* is the diffusional exponent (*n* = 0.5 for Fickian mechanism). This equation can be modified for spherical geometry to the following equation, as we have reported before [36,37]:

(1) $$\frac{M_{t}}{M_{\infty }}=6{\left(\frac{Dt}{\pi R^{2}}\right)}^{\frac{1}{2}}$$

where *M_t_/M_∞_* represents the fraction of released VEGF at time *t*, *D* is the diffusion coefficient of the VEGF, and *R* is the radius of the particles (8 µm). The calculated values for the diffusion coefficient (Figure 2D) and the cumulative amount of released VEGF after 24 h as indicator of burst released VEGF (Figure 2E) are shown for alginate and alginate-heparin. On the basis of these results, presence of heparin prolongs release due to increased affinity toward the microparticles.

**Figure 3:**
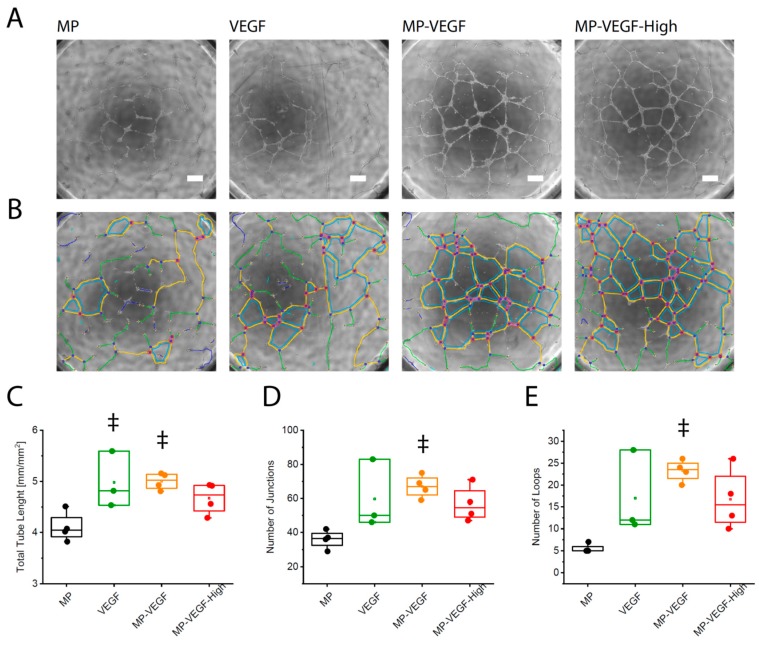
2D capillary network formation. Capillary-like network formation by endothelial cells on growth factor-reduced Matrigel affected by addition of microparticles releasing VEGF-A. (**A**) Morphology of vascular networks after 24 h and (**B**) evaluation with the Angiogenesis Analyzer ImageJ plugin; red dots indicate junctions, blue lines indicate loops, scale bar: 300 µm. MP: microparticles, MP-VEGF: microparticles releasing VEGF-A (50 ng/mL initial loading), MP-VEGF-H: microparticles releasing high concentration of VEGF-A (100 ng/mL initial loading). (**C**) Total vascular tube length. (**D**) Number of junctions. (**E**) Number of loops, ‡ indicates significant difference comparing to samples containing microparticles alone (*p* < 0.05) evaluated by one-way ANOVA.

**Figure 4:**
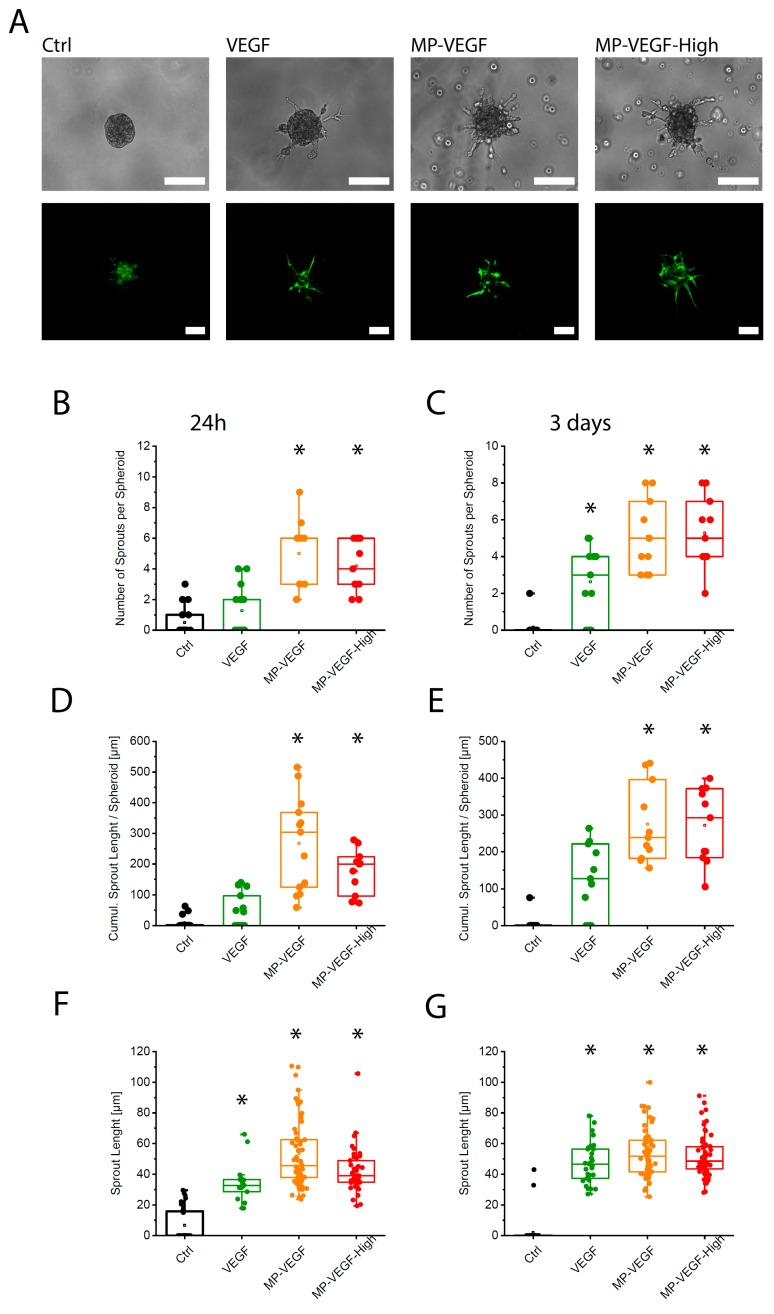
VEGF release accelerate spheroids sprouting in 3D. (**A**) The effect of microparticles releasing VEGF-A on endothelial cell sprouting from spheroids in 3D microenvironments. Morphology of endothelial cell sprouts in bright-field and fluorescence microscopy images, scale bar: 100 µm. Ctrl: HUVEC spheroids grown in EGM-2 medium, VEGF: HUVEC spheroids stimulated with 50 ng/mL VEGF-A, MP-VEGF: microparticles releasing VEGF-A (50 ng/mL initial loading), MP-VEGF-H: microparticles releasing high concentration of VEGF-A (100 ng/mL initial loading). Number of sprouts per spheroid after (**B**) 24 h and (**C**) 3 days. Cumulative sprout length after (**D**) 24 h and (**E**) 3 days. Length of individual sprouts after (**F**) 24 h and (**G**) 3 days. * indicates significant difference comparing to Ctrl (*p* < 0.001) evaluated by one-way ANOVA.

The effect of microparticle (MP)-released VEGF on endothelial cell behavior in 2D (Figure 3) and 3D (Figure 4) microenvironments was evaluated after 24 h or 3 days. After 24 h incubation, HUVECs, seeded as single cells on growth-factor reduced Matrigel, formed capillary-like structures with significantly increased total tube length when incubated with microparticles releasing 50 ng of VEGF per milliliter compared to MP alone (Figure 3). Comparable results were obtained after endothelial cell stimulation with 50 ng/mL VEGF. VEGF-loaded microparticles (MP-VEGF) also increased number of junctions and loops in the capillary-like network (Figure 3D,E). No additional improvement was observed when HUVECs were incubated with MP releasing a higher concentration of VEGF (100 ng/mL initial loading).

To test the impact of MP-VEGF on angiogenesis in the 3D microenvironment, spheroids of endothelial cells with a size of 500 cells per spheroid were formed and embedded in the growth-factor reduced Matrigel. Figure 4A shows the morphology of endothelial cell sprouts growing from a spheroid after 3 days of culture. A significantly higher number of sprouts were observed in samples stimulated by VEGF-A-loaded microparticles compared to the control and to the samples containing VEGF-A alone in both time points (Figure 4B,C). Cumulative sprout length per spheroid, that is, the total length of all sprouts growing from a spheroid, was also significantly higher in samples containing MP-VEGF (Figure 4D,E). No significant difference was observed between the two concentrations of VEGF tested. All samples containing VEGF showed an increased length of individual sprouts compared to the unstimulated control (Figure 4F,G), but overall MP-VEGF were the most potent stimulators of angiogenesis in the 3D microenvironment. Although VEGF is a potent growth factor to initiate angiogenesis, formation of mature and stable vascular network is dependent on the presence of multiple growth factors [38,39]. In future studies, we will examine the co-delivery of multiple growth factors such as VEGF and platelet-derived growth factor (PDGF) [40] to not only to promote angiogenesis but also to help maturation of formed blood vessels.

**Figure 5:**
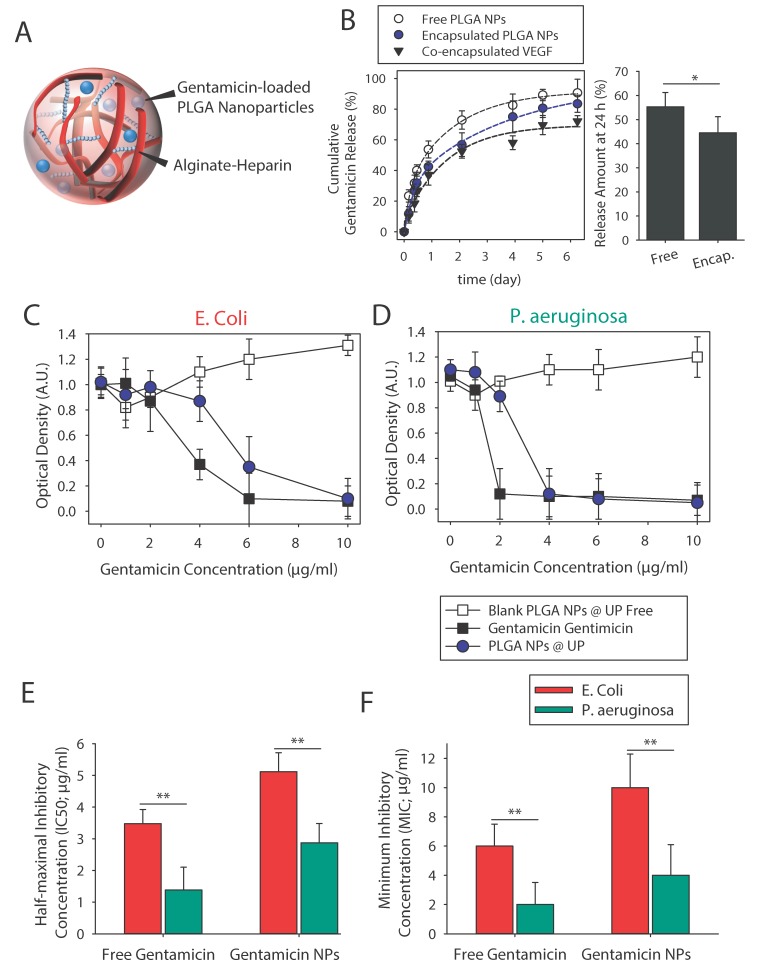
Evaluation of gentamicin release. (**A**) Gentamicin-loaded poly(lactic-co-glycolic) acid (PLGA) nanoparticles (NPs) encapsulated in alginate-heparin microparticles. Estimated amount of released gentamicin after 24 h. (**B**) Cumulative release profiles of gentamicin from free PLGA NPs and from microparticle-encapsulated PLGA NPs. Cumulative release profiles of co-encapsulated VEGF from alginate-heparin microparticles are also shown here. Analysis of the ability of gentamicin PLGA NPs to inhibit the growth of (**C**) *Escherichia coli* and (**D**) *Pseudomonas aeruginosa* as determined by measurement of absorbance at 600 nm. Estimated (**E**) half-maximal inhibitory (IC50) and (**F**) minimum inhibitory concentrations (MIC) values for free and PLGA-encapsulated PLGA NPs (average ± SD, *n* = 3). * and ** indicate significant difference of *p* < 0.05 and *p* < 0.01, respectively, as evaluated by one-way ANOVA. The lines in the graphs are a guide for the eye.

Besides delivering growth factors that promote chronic wound healing through neovascularization, our microparticles are able to simultaneously release antibacterial agents that prevent potential infections. Here, gentamicin was chosen for its well-known clinical benefits [41] as an antibiotic agent co-encapsulated into the alginate-based microparticles. Gentamicin can be loaded without further modification, or it can be encapsulated into nanoparticles to obtain tighter control over its release rate (Figure 5A). Here, we used a water-in-oil-in-water double emulsification evaporation technique to form poly(lactic-co-glycolic) acid (PLGA) nanoparticles (NPs). The average hydrodynamic size of the PLGA NPs was measured by DLS, and found it to be 221.5 ± 10.5 nm with a polydispersity index of 0.12 ± 0.03 and surface charge of 0.8 ± 0.3 mV. We found that our PLGA NPs can encapsulate 28.7 ± 3.6 μg/mg PLGA. We also analyzed gentamicin release from PLGA NPs (Figure 5B). On the basis of the release profile, 55% of gentamicin was released from PLGA NPs after 24 h. It should also be noted that as the microparticle size will be controlled by size and geometry of the formed droplet during the droplet generation process, presence of PLGA NPs did not affect the size of the resulting microparticles. Encapsulation of the NPs inside alginate-based microparticles will not significantly alter the release profile after 4 days (*p* > 0.05), but it will reduce the released amount at earlier time points (< day 3). This might be due to the temporary entrapment of this drug in the alginate microgels.

We tested the antibacterial properties of gentamicin-loaded particles against two well-known bacteria strains that often contribute to wound infection, *P. aeruginosa* and *E. coli.* Bacterial growth was measured after a 24 h incubation of the mentioned bacteria with varying concentrations (up to 10 µg/mL) of free gentamicin and gentamicin-loaded particles (Figure 5C,D). Free gentamicin was able to inhibit bacterial growth due to the sustained release feature of the NPs. The unloaded NPs showed no inhibition of bacterial growth, underlining that the effects were caused by the gentamicin only. The calculated half-maximal inhibitory (IC_50_) and minimum inhibitory (MIB) concentrations are shown in Figure 5E,F. The obtained values are in accordance with results reported previously [21,22,42]. 

## 4. Conclusions

In summary, we developed a microfluidic-assisted method to fabricate microparticles with encapsulated nanoparticles. These particles offer a modular platform to deliver a wide range of proteins as well as small molecule therapeutics. Co-encapsulation of antibiotic-loaded nanoparticles inside the growth factor-releasing microparticles proved to be a useful combinatorial drug delivery system that was not only able to fight the wound infection but also to help tissue healing by increasing angiogenesis. This approach offers countless possibilities for synthesis of other types of tailored, drug-loaded, nano-in-microparticles with various properties that may have broad applications in regenerative medicine and tissue engineering.
